# Quantifying bacterial concentration in water and sand media during flow-through experiments using a non-invasive, real-time, and efficient method

**DOI:** 10.3389/fmicb.2022.1016489

**Published:** 2022-12-23

**Authors:** Xiaoming Zhang, Fengxian Chen, Liqiong Yang, Fucang Qin, Jie Zhuang

**Affiliations:** ^1^College of Desert Control Science and Engineering, Inner Mongolia Agricultural University, Hohhot, China; ^2^Key Laboratory of Pollution Ecology and Environmental Engineering, Institute of Applied Ecology, Chinese Academy of Sciences, Shenyang, Liaoning, China; ^3^College of Forestry, Inner Mongolia Agricultural University, Hohhot, China; ^4^Department of Biosystems Engineering and Soil Science, Center for Environmental Biotechnology, The University of Tennessee, Knoxville, TN, United States

**Keywords:** bacterial transport, porous media, bioluminescent imaging, non-invasive quantification, bioluminescent count

## Abstract

Monitoring the dynamics of bacteria in porous media is of great significance to understand the bacterial transport and the interplay between bacteria and environmental factors. In this study, we reported a non-invasive, real-time, and efficient method to quantify bioluminescent bacterial concentration in water and sand media during flow-through experiments. First, 27 column experiments were conducted, and the bacterial transport was monitored using a real-time bioluminescent imaging system. Next, we quantified the bacterial concentration in water and sand media using two methods—viable count and bioluminescent count. The principle of the bioluminescent count in sand media was, for a given bioluminescence image, the total number of bacteria was proportionally allocated to each segment according to its bioluminescence intensity. We then compared the bacterial concentration for the two methods and found a good linear correlation between the bioluminescent count and viable count. Finally, the effects of porous media surface coating, pore water velocity, and ionic strength on the bioluminescent count in sand media were investigated, and the results showed that the bioluminescence counting accuracy was most affected by surface coating, followed by ionic strength, and was hardly affected by pore water velocity. Overall, the study proved that the bioluminescent count was a reliable method to quantify bacterial concentration in water (10^6^ to 2 × 10^8^ cell mL^−1^) or sand media (5 × 10^6^-5 × 10^8^ cell cm^−3^). This approach also offers a new way of thinking for *in situ* bacterial enumeration in two-dimensional devices such as 2D flow cells, microfluidic devices, and rhizoboxes.

## Highlights

1. We proposed a method to quantify bacterial concentration in sand media.
2. This method avoided reliance on calibration curves.
3. The method's accuracy was affected by sand surface coating and ionic strength.

## 1. Introduction

In terrestrial ecosystems, bacteria may enter the soil in various ways, such as manure fertilization, reclaimed water irrigation, and sludge application. A better understanding of bacterial transport in the soil is essential to reduce bacterial contamination (Pachepsky et al., 2006; Bradford et al., 2013). Flow-through experiments (e.g., column experiment and flow cell experiment) were usually used to examine the interplay between various environmental factors and bacterial transport, in which breakthrough curves were easily obtained for the description of transport behaviors (Tufenkji, 2007; Hron et al., 2015). However, obtaining bacterial deposition profiles in soil column or flow cell was difficult, mainly attributed to two reasons. One is the invasiveness of the sampling process. The soil columns or flow cells needed to be cut or opened for sampling, leading to interruption of the bacterial transport process (Bai et al., 2016; Wu et al., 2018). The other is the irreversible adsorption of bacteria, causing incomplete extraction and measurement (Zhong et al., 2017; Powelson and Gerba, 2018). Moreover, the abovementioned sampling and extraction procedures are time-consuming and laborious. Therefore, a simple, non-invasive, real-time, and efficient method is essential for the quantification of bacterial deposition profiles in flow-through experiments.

Over the past three decades, fluorescent or bioluminescent methods have been widely used to monitor cell activity due to the development of green fluorescent proteins in cell biology studies (Tsien, 1998). Fluorescent or bioluminescent reporter strains were the most common tools to monitor the dynamics of bacteria in transparent or translucent porous media such as quartz sand, polydimethylsiloxane, and Nafion particles (Uesugi et al., 2001; Jost et al., 2015; Liu et al., 2021; Yang J. Q. et al., 2021). However, in current methods, there are two main limitations to bacterial enumeration in porous media using calibration curves. One is the cumbersome steps to build a calibration curve. For example, Jost et al. (2015) established a non-linear calibration curve between bacterial concentration and fluorescence intensity to quantify the concentration of *Escherichia coli* in unsaturated sands. Although the method was reliable, the cumbersome construction of the calibration curve limited its promotion and use. The other limitation is the instability of the calibration curve. The bioluminescence intensity was greatly affected by nutrients and oxygen availability, causing the calibration curve to become inaccurate over time (Uesugi et al., 2001; Yarwood et al., 2002; Bozorg et al., 2015). Therefore, a method that does not require a calibration curve is worth exploring.

For bacteria in water, we determined the bacterial concentration using a real-time calibration curve; for bacteria in sand media, we proportionally allocated total bacteria to each segment in each bioluminescence image of sand columns according to its bioluminescence intensity. The main purpose of this study was to evaluate the feasibility and accuracy of the bioluminescent count during flow-through experiments. Twenty-seven column experiments were conducted in this study, and the bacteria in water (effluent) and sand media (sand column) were enumerated using two methods: viable count and bioluminescent count. The accuracy of the bioluminescent count was compared with the viable count, and the effects of porous media surface coating (clean sand, humic acid coating, or iron oxide coating), pore water velocity, and ionic strength were examined.

## 2. Materials and methods

### 2.1. Bacterial strain and growth conditions

*Escherichia coli* 652T7, used in this experiment, is a luminescent reporter strain that contains a plasmid that carries the *Photorhabdus luminescens* luxCDABE gene (provided by the University of Tennessee's Center for Environmental Biotechnology in Knoxville, TN, USA). *E. coli* 652T7 can emit a 490 nm light signal that can be captured by a cooled charge-coupled device (CCD) camera. The strain was cultivated in a sterile 250 mL Erlenmeyer flask containing 100 mL Luria Broth growth medium until the early stationary phase (~13 h at 37 °C, on an orbital shaker at 160 rpm). The early stationary phase cells ensured a relatively constant bacterial concentration and metabolically active state. The bacterial culture was harvested by centrifugation (5,000 g for 10 min at 25 °C). The supernatant was removed, and the cells were resuspended in 50 mL NaCl solution with different ionic strengths (2, 20, and 100 mM). The washing process was repeated three times. The washing process removed all the adherent culture medium and produced a stable bacterial suspension for subsequent experiments. The final bacterial suspension was diluted using NaCl solution to an optical density at 600 nm (OD_600_) of ~0.4 (equivalent to ~ 2 × 10^8^ CFU mL^−1^).

### 2.2. Porous media

Medium-sized quartz sand (425–500 μm; Shanghai Maclean Biochemical Technology Co., Ltd., China) was used in this experiment. The quartz sand was washed sequentially using 10 mM HCl, 10 mM NaOH solution, and ultrapure water and then dried in an oven at 110 °C for use. Three types of quartz sands with different surface properties were prepared: clean sand, humic acid-coated sand, and iron oxide-coated sand. Humic acid and iron oxide were used as coating materials because they are common substances found in natural soils (Xing et al., 2020). Clean sand was obtained after the abovementioned washing procedure; humic acid-coated sand was prepared using the method described by Zhuang and Jin (2003), and the concentration of humic acid was 0.481 mg g^−1^; iron oxide-coated sand was prepared using the method described by Benjamin et al. (1996), and the concentration of iron oxide was 0.064 mg g^−1^. The bulk densities of clean sand, humic acid-coated sand, and iron oxide-coated sand were 1.72, 1.69, and 1.61 g cm^−3^, respectively.

### 2.3. Column experiments

**Figure 1:**
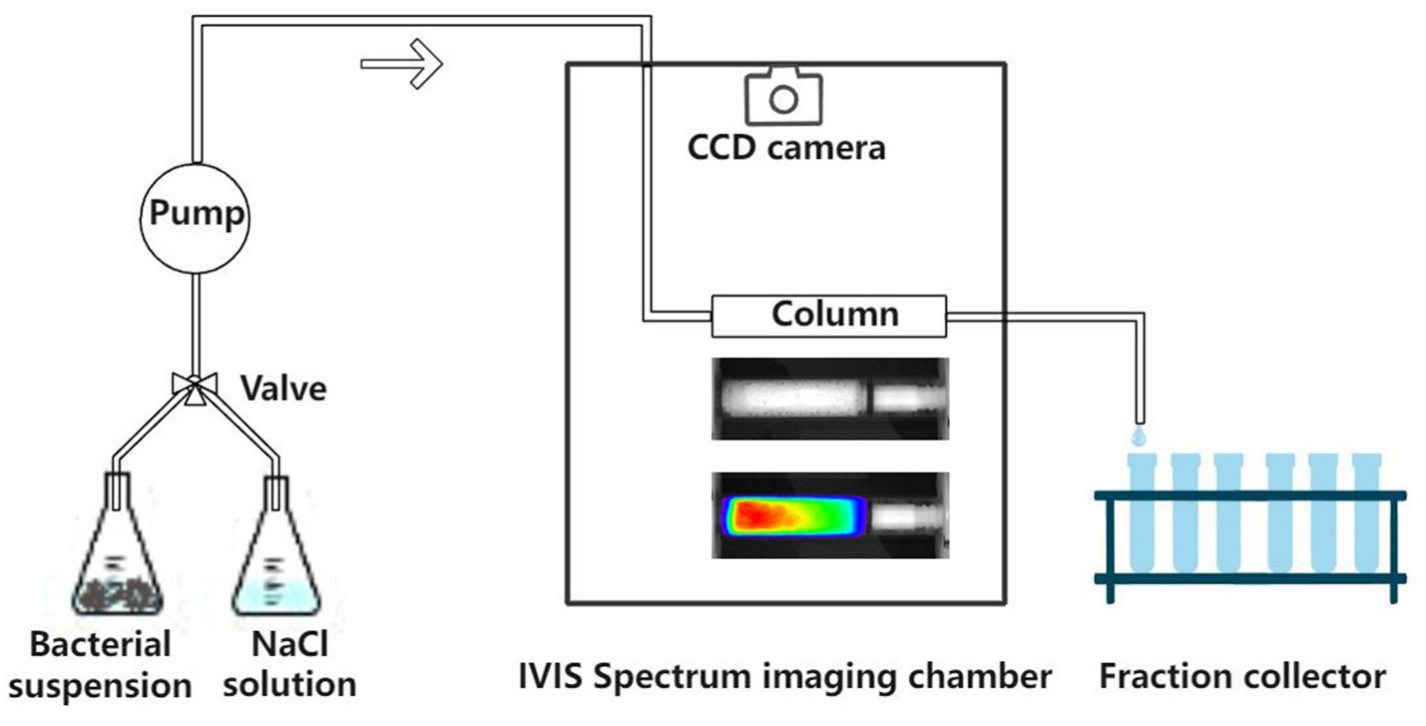
Schematic diagram of the experimental setup.

A glass column (1.33 cm inner diameter and 6 cm length) was packed with dry quartz sand and saturated from the bottom by pumping 20 pore volumes of background solution (NaCl solution) through the column at a discharge rate of Q = 1 mL min^−1^. After this, the column was placed horizontally in the IVIS Spectrum imaging chamber (PerkinElmer). Imaging of the sand column using the IVIS Spectrum system was performed following the method provided by Zhuang et al. (2020). The experimental setup is shown in Figure 1. A pulse input (four pore volumes) of bacterial suspension (OD_600_ = 0.4) was applied to the column inlet at a specific discharge rate, followed by two pore volumes of background solution. The CCD camera captured the bioluminescence emission from the sand column at one pore volume intervals. An example is shown in **Figure 3A**. The effluent was collected in glass test tubes with a fraction collector at 0.25 pore volume intervals, and the bacteria were enumerated by their bioluminescence intensities in a 96-well black plate (Yang L. et al., 2021). To verify the validity of the bioluminescent count for the effluent, four columns were chosen from 27 and the bacterial concentration in the effluent was determined using the viable count and bioluminescent count. Moreover, in addition to three different types of sands, three pore water velocities (3.55, 10.56, and 21.30 cm h^−1^) and three ionic strengths of the background solution (2, 20, and 100 mM NaCl) were used as independent variables to test their effects on the bioluminescence imaging. At the end of each column experiment, the sands were extracted and cut into six segments. The sands from each segment were collected in 30 mL flasks and mixed with 2 mM NaCl (1:30 w/v), and the bacteria were extracted from the sands.

### 2.4. Quantification of bacterial concentration

A preliminary test was performed in 96-well black plates to examine the relationship between bacterial concentration and bioluminescence intensity in water and saturated sand. The bacterial suspension was diluted to 10 different concentrations (OD_600_ ranging from 0 to 1.2). For bacteria in water, 200 μL of bacterial suspension was added to a 96-well black plate. For bacteria in sand, 60 μL of bacterial suspension was added to a well, and then 0.4 g of sand was added. Due to the capillary effect, the sand was saturated from the bottom with bacterial suspension after 10–15 min. The 96-well black plate was placed in the IVIS Spectrum imaging chamber, and the bioluminescence intensity was captured. Each measurement comprised of three replicates.

**Figure 2:**
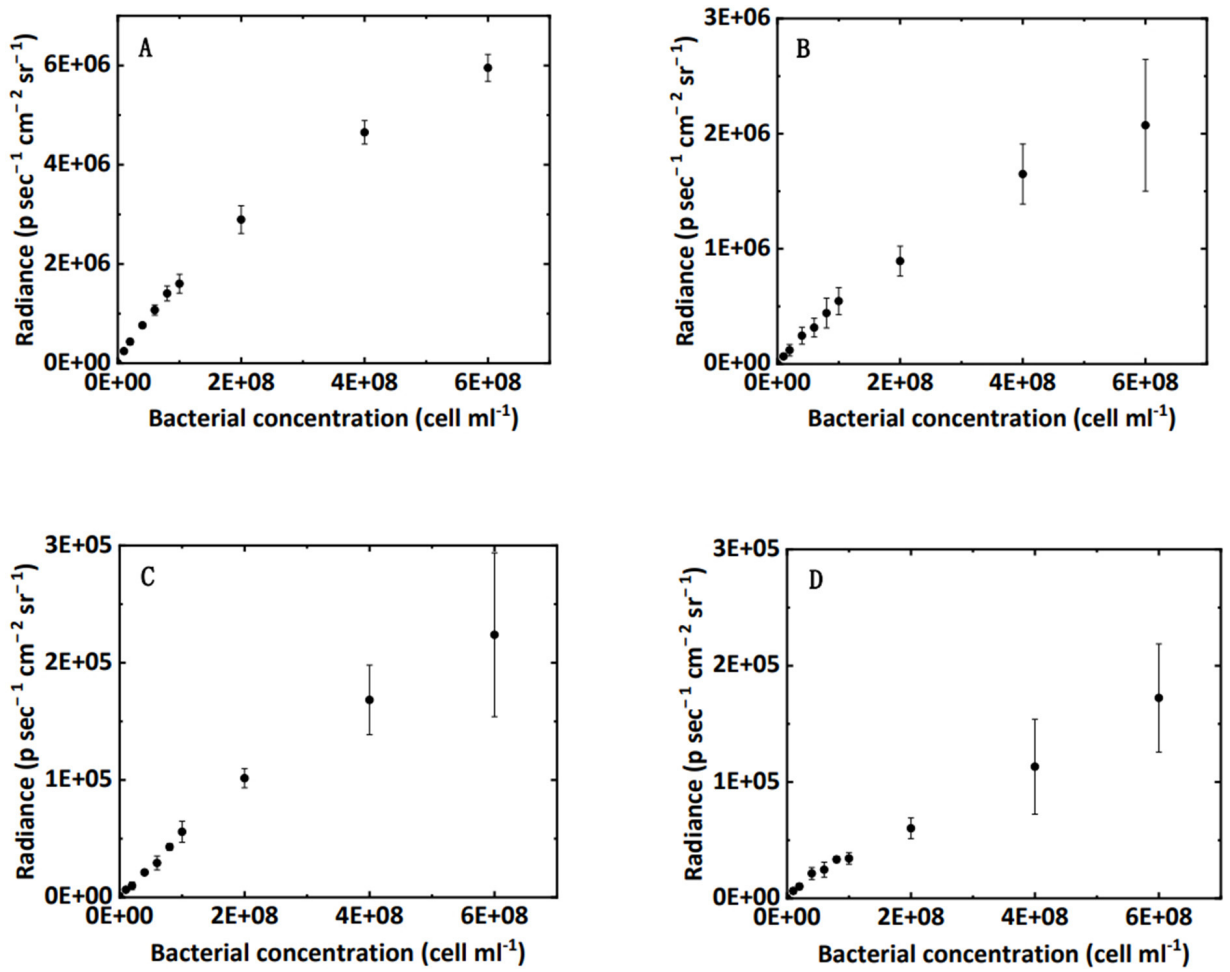
Correlation between bacterial concentration in water **(A)**, clean sand **(B)**, humic acid-coated sand **(C)**, and iron oxide-coated sand media **(D)** and the luminescence intensity in 96-well black plates.

**Figure 3:**
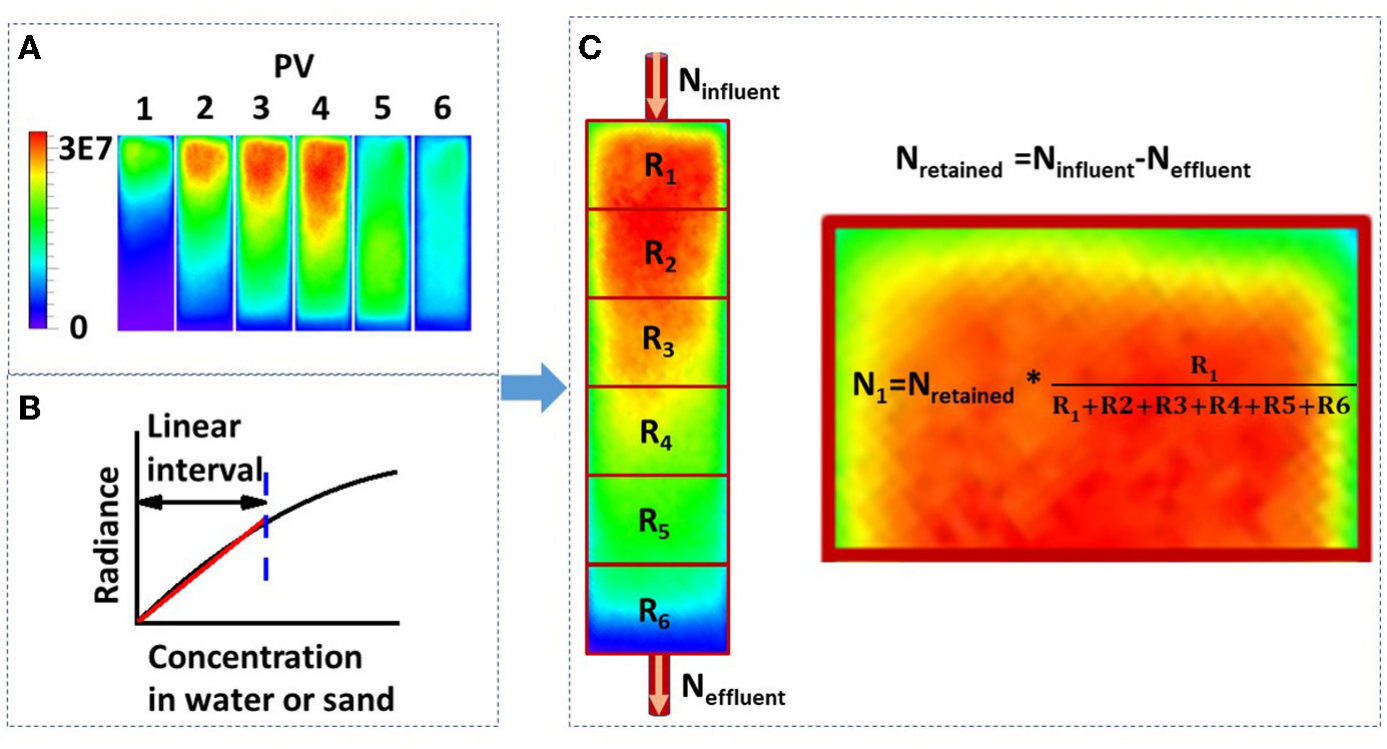
**(A)** Luminescence images of bacterial transport in a sand column captured using a real-time bioluminescent imaging system (PV, pore volume; PV1-4, the influent was the bacterial suspension; PV5-6, the influent was the background solution; the scale bar represents the bioluminescence intensity, i.e., radiance); **(B)** schematic diagram of the correlation between bacterial concentration in water or sand media and the luminescence intensity; and **(C)** method for calculating the bacterial number from the luminescence intensity (N_influent_, total bacterial number in the influent; N_effluent_, total bacterial number in the effluent; N_retained_, total bacterial number retained in the sand column; R_1_-R_6_, mean luminescence intensity from segment 1 to 6; N_1_, total bacterial number retained in the first segment).

Two methods—viable count and bioluminescent count—were used to quantify the bacterial concentration in sand columns (Reed and Reed, 1948). The viable count method was used to quantify the extracted bacteria from sand: 10-fold serial dilutions were conducted in sterile PBS (pH 7.4), and three replicates of 100 μL aliquots from selected dilutions were dispensed onto LB agar plates. The plates were incubated at 37 °C for 24 h. The bacterial concentration was calculated as follows: (number of colonies × total dilution factor)/spot volume. The schematic diagram of the bioluminescent count method is shown in Figure 3C, and the procedures were as follows: first, the total retained bacteria number was calculated from the mass balance, that is, the total retained bacteria equal to the total influent bacteria minus the total effluent bacteria; second, the average bioluminescence intensity of each segment was calculated using the Living Images software package (PerkinElmer); third, the total number of bacteria was proportionally allocated to each segment according to the ratio of their bioluminescence intensities. In brief, a linear correlation was proven, as shown in Figure 2, and this was used to calculate the bacterial concentration in Figure 3C. It should be mentioned that the bacteria in this study were harvested at the early stationary phase and no nutrients were added to bacterial suspension; therefore, it was assumed that, within several hours (e.g., 12 h), bacteria did not grow or die.

### 2.5. Statistical analysis

The correlation between the bacterial concentration from the bioluminescent count and that from the viable count was fitted by a straight line: y = x. The performance of regression was evaluated using two parameters in Origin 2018 software: (1) coefficient of determination (R^2^) and (2) normalized root-mean-square deviation (NRMSD)—normalized by the mean value of bacterial concentration from the bioluminescent count.

## 3. Results and discussion

### 3.1. The rationale for the bioluminescent count method

The relationship between bacterial concentration and bioluminescence intensity in water, clean sand, humic acid-coated sand, and iron oxide-coated sand in a 96-well black plate is shown in Figure 2. Within a certain concentration range (under 2 × 10^8^ cell cm^−3^), the interval could be approximated by a linear increase. A schematic diagram describing this relationship is shown in Figure 3B. Numerous studies showed a linear or near-linear relationship between the bacterial concentration in water and the bioluminescence intensity (Bozorg et al., 2015; Yang L. et al., 2021; Xu et al., 2022). However, we extended the linear relation to bacteria enumeration in sand media. The results proved the theory for the bioluminescent count method: assuming that the bacterial concentration is X1 and the corresponding radiance is Y1; if the bacterial concentration is two times X1, the corresponding radiance should be two times Y1. Conversely, if the radiance observed is two times Y1 in sand media, the corresponding bacterial concentration should be two times X1. Based on this rationale, a method to calculate bacterial concentration using bioluminescence was proposed (Figure 3C).

### 3.2. Bioluminescence in effluent

Using a calibration curve (e.g., Figure 2A) to calculate the bacterial concentration in water is nothing new. Notably, for the effluent of the columns, the bioluminescence intensity may weaken over time owing to the consumption of energy (Yang et al., 2020; Ofer et al., 2021). Therefore, the most important note here is that the bacterial concentration must be calculated using the calibration curve on the same bioluminescence image, which will ensure a valid and reliable bioluminescent count in the effluent.

**Figure 4:**
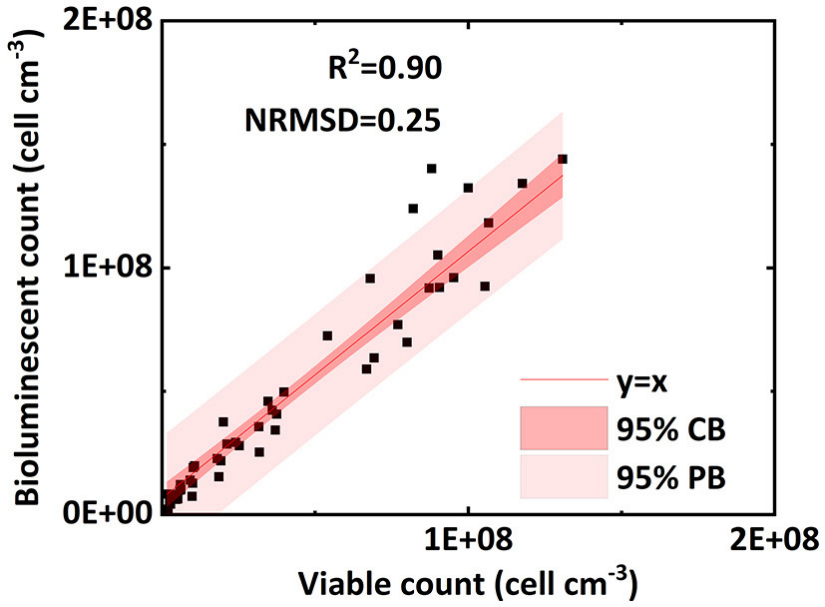
Linear correlation of bacterial concentration (cell ml^−1^) between the bioluminescent count and viable count for bacteria in the effluent of column experiments (NRMSD, normalized root-mean-square deviation; CB, confidence band; PB, prediction band).

Figure 4 verifies the performance of the bioluminescent count method in the effluent. Figure 4 shows an excellent linear correlation (R^2^ = 0.90, NRMSD = 0.25) between the bioluminescent count and viable count for bacteria in the effluent over the concentration range 10^6^ to 2 × 10^8^ cell mL^−1^. In the preliminary test (Figure 2A), the linear interval of bacterial concentration in water ranged from 10^7^ to 2 × 10^8^ cell mL^−1^. Figure 4 extends the lower limit (10^7^ cell mL^−1^) to a lower concentration (10^6^ cell mL^−1^), that is, a wider applicable range. For a bacterial concentration higher than 2 × 10^8^ cell mL^−1^, the bacterial bioluminescence may be subject to oxygen availability, causing a non-linear correlation between the bacterial concentration in water and their bioluminescence (Figure 2A). This phenomenon was also extensively observed in the literature (Uesugi et al., 2001; Bozorg et al., 2015; Wilson et al., 2018; Tanet et al., 2019). However, in this study, we only focused on the linear interval (10^6^ to 2 × 10^8^ cell mL^−1^), which was the premise that allowed us to simplify the bioluminescent count. Moreover, the bacterial concentration used in previous flow-through experiments mostly ranged from 10^7^ to 10^8^ cell mL^−1^ (Sepehrnia et al., 2018; He et al., 2021; Yang L. et al., 2021), which was within the linear interval. Therefore, the applicable range (10^6^ to 2 × 10^8^ cell mL^−1^) of the bioluminescent count was sufficient for most cases.

### 3.3. Bioluminescence in sand

**Figure 5:**
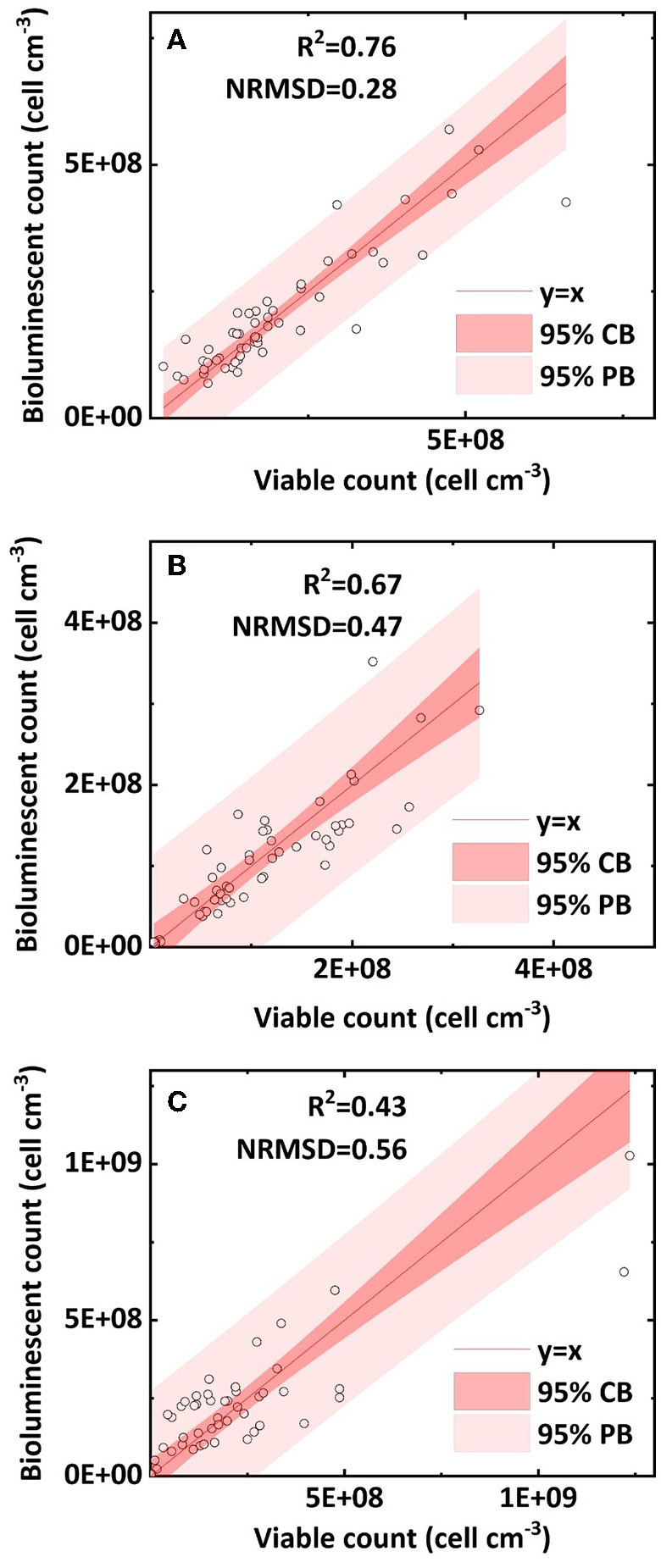
Linear correlation of bacterial concentration (cell cm^−3^) between the bioluminescent count and viable count in sand columns: **(A)** clean sand; **(B)** humic acid-coated sand; and **(C)** iron oxide-coated sand (NRMSD, normalized root-mean-square deviation; CB, confidence band; PB, prediction band).

We compared the retained bacterial concentration determined by the bioluminescent count and viable count in clean sand, humic acid-coated sand, and iron oxide-coated sand. The R^2^ values of their linear regressions were 0.76, 0.67, and 0.43, respectively; the NRMSD values were 0.28, 0.47, and 0.56, respectively (Figures 5A–C). The above statistical parameters indicated that the bioluminescent count for clean sand had better accuracy than that for humic acid-coated sand or iron oxide-coated sand, attributed to the light transmissibility. A preliminary measurement (data not shown) determined the order of light transmissibility among the three types of sands, that is, highest for clean sand, followed by humic acid-coated sand, and lowest for iron oxide-coated sand. The different light transmissibilities apparently affected the total light captured by the CCD camera of the bioluminescent imaging system (Yarwood et al., 2006; Rockhold et al., 2007; Bozorg et al., 2015). This was also confirmed by the quality of bioluminescence images obtained from our experiments: clean sand presented the best quality bioluminescence images, followed by humic acid-coated sand, and iron oxide-coated sand. Therefore, precision may need to be sacrificed when applying this bioluminescent count method to colored porous media.

The bacterial concentration in clean sand ranged from 6 × 10^7^ to 5 × 10^8^ cell cm^−3^; that in humic acid-coated sand ranged from 4 × 10^7^ to 3 × 10^8^ cell cm^−3^; and that in iron oxide-coated sand ranged from 5 × 10^6^ to 10^9^ cell cm^−3^. The reason for the different bacterial concentration ranges in the three sands was that different surface coatings affected bacterial transport. Previous studies showed that humic acid facilitated bacterial transport in sands because of adsorption site competition, whereas iron oxide greatly reduced bacteria transport in sands because of its positive surface charge (Zhang et al., 2010; Yang et al., 2012). However, it is still not clear whether the accuracy of the bioluminescent count is affected by bacterial transport and retention in sand columns. Overall, most bacterial concentrations within the 95 % prediction band ranged from 5 × 10^6^ to 5 × 10^8^ cell cm^−3^. Uesugi et al. (2001) used a CCD camera to capture the bioluminescence during the induction phase of *Pseudomonas fluorescens* in saturated sand, at bacterial concentrations ranging from 10^6^ to 10^8^ cell mL^−1^. Jost et al. (2015) built a calibration curve and successfully quantified green fluorescent protein-labeled *E. coli* in unsaturated sand at bacterial concentrations ranging from 5 × 10^7^ to 6 × 10^8^ cell cm^−3^. In our study, the applicable concentration range of the bioluminescent count (5 × 10^6^ to 5 × 10^8^ cell cm^−3^) can be regarded as a broad and practical range, and within this applicable range, the bioluminescent count can be regarded as a reliable method. More importantly, it removes the dependence on a bacterial induction phase or a calibration curve and can be applied simply and conveniently.

### 3.4. Effect of pore water velocity and ionic strength

**Figure 6:**
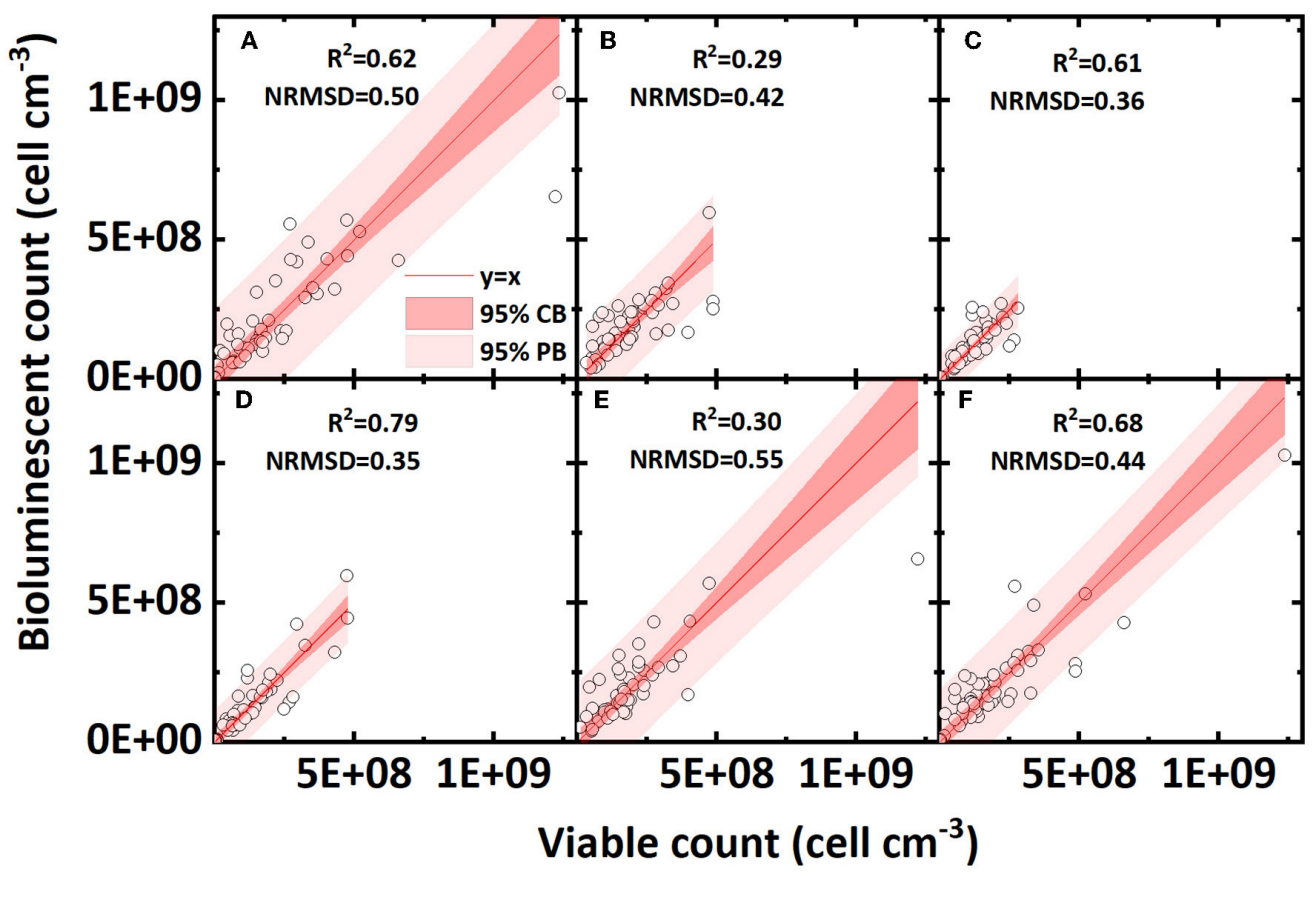
Linear correlation of bacterial concentration (cell cm^−3^) between the bioluminescent count and viable count in sand columns at different conditions (pore water velocity: **(A)** low velocity, 3.55 cm h^−1^; **(B)** medium velocity, 10.56 cm h^−1^; **(C)** high velocity, 21.3 cm h^−1^; ionic strength of background solution: **(D)** low ionic strength, 2 mM NaCl; **(E)** medium ionic strength, 20mM NaCl; **(F)** high ionic strength, 100 mM NaCl; NRMSD, normalized root-mean-square deviation; CB, confidence band; PB, prediction band).

The effect of pore water velocity on the accuracy of the bioluminescent count is shown in Figures 6A–C. A higher water flow velocity mainly increased the shear force in pores and thus reduced the bacterial mechanical straining (Bradford et al., 2006; Yang L. et al., 2021). Low and high velocities presented similar R^2^ values, and a medium velocity exhibited a lower R^2^ value. Overall, the pore water velocity does not have an apparent effect on the accuracy of the bioluminescent count.

Different ionic strengths reflect the effect of electrostatic force on the accuracy of the bioluminescent count and the results are shown in Figures 6D–F. As the bacteria carry a negative charge on their surfaces, for surface negative-charged sands (clean sand and humic acid-coated sand), the increased ionic strength reduced the electrostatic repulsion and compressed the electrical double layer, thus greatly increasing the bacterial retention in sands. For surface positive-charged sands (iron oxide-coated sand), the effect of ionic strength was reversed; nevertheless, most bacteria were already tightly adsorbed on sand surface so that the effect of ionic strength was minor (Mills et al., 1994; Yee et al., 2000; Zhang et al., 2010). Comparing the regressions of three ionic strengths, a low ionic strength presented the highest R^2^ value and the lowest NRMSD value, indicating that a lower ionic strength may contribute to a better bioluminescent count. Considering that the ionic strength had different effects for surface positive- and negative-charged sands, we also calculated the R^2^ value of the linear regression for only negative surface sands (clean sand and humic acid sand; figures not shown). In surface negative-charged sands containing 2, 20, and 100 mM NaCl, the R^2^ values were 0.87, 0.85, and 0.53, respectively, which shows the same regularity as indicated in Figures 6D–F, that is, a lower ionic strength improves the accuracy of the bioluminescent count.

The effect of ionic strength and surface charge on the bioluminescent counting accuracy may be explained by the bacterial adsorption. Generally, bacteria were retained in saturated sand in two phases: liquid or solid (Zhong et al., 2017). For surface negative-charged sands, the enhanced ionic strength reduced the repulsive force between the bacteria and sand, and thus, more bacteria were adsorbed in the solid phase (Israelachvili, 2011). Bacterial aggregation on solid surfaces may amplify the effect of solid phase shading and may reduce the accuracy of the bioluminescent count. This may also explain, to some extent, why iron oxide-coated sand exhibited the lowest accuracy (most bacteria were adsorbed in the solid phase owing to its positive surface charge). Overall, the ionic strength and surface charge affected bacterial adsorption in the solid phase, which most likely affected the accuracy of the bioluminescent count.

### 3.5. Limitations of the bioluminescent count method

We must admit three main limitations of this method. The first is that the total amount of retained bacteria in the sand columns should be known in order to calculate the bacterial concentration in the sand media. The second is that water saturation is an important factor in light transmissibility (Uesugi et al., 2001; Jost et al., 2015), and therefore, further research is needed to improve this method in variably saturated porous media. The third is that we did not add nutrients to the bacteria, and only use this linear relation to calculate the bacterial concentration—the tradeoff is that this method can only be used inside a short period of time (e.g., within 1 day).

In addition, the diameter of the column strongly affected the light detection. A preliminary test showed that the CCD camera only captured light from the first 4–5 mm of the surface of the quartz sand. In a one-dimensional experiment, the light on the surface may proportionally represent the total bioluminescence of the whole depth. Therefore, the diameter of the column affected the light detection, but had only a minor effect on the accuracy of the bioluminescent count. However, this also indicates that our bioluminescent count method can only be used in one- or two-dimensional experiments, but not three-dimensional experiments.

## 4. Conclusion

The bioluminescent count method has been proved to be a reliable method to quantify bioluminescent bacterial concentration in saturated sand media. The accuracy of the bioluminescent count was affected by the sand surface coating (light transmissibility and surface charge), partly affected by the ionic strength (bacterial adsorption), and almost unaffected by the pore water velocity. Compared to other common methods (e.g., viable count and microscope count), it provides a simplified, non-invasive, real-time, and efficient way to investigate bacterial transport and their interplay with various environmental factors in sand media. The method also shows potential for bacterial enumeration using bioluminescent imaging in two-dimensional flow-through experiments (e.g., 2D flow cell and microfluidic device) or two-dimensional devices (e.g., rhizobox).

## Data availability statement

The raw data supporting the conclusions of this article will be made available by the authors, without undue reservation.

## Author contributions

XZ performed the experiments and analyzed the data. FC analyzed the data and drafted the article. JZ designed the experiments. LY, FQ, and JZ reviewed the article. All authors contributed to the article and approved the submitted version.
